# Activation of MAP kinases by green leaf volatiles in grasses

**DOI:** 10.1186/s13104-017-3076-9

**Published:** 2018-01-29

**Authors:** James E. Dombrowski, Ruth C. Martin

**Affiliations:** USDA-ARS, National Forage Seed Production Research Center, 3450 SW Campus Way, Corvallis, OR 97331-7102 USA

**Keywords:** Grass, Green leaf volatiles, Kinases, *Lolium*, MAPK, Volatiles, Wounding

## Abstract

**Objective:**

Previously we have shown that mechanical wounding and volatiles released from cut grass, activated a 46 and 44 kDa mitogen-activated protein kinase (MAPK) in the model grass species *Lolium temulentum* (*Lt*). MAPKs play an important role as signal relays that connect incoming stress signals and stress responses. Since green leaf volatiles (GLV) are released during wounding, we wanted determine if specific compounds contained in the GLV mixture or if GLV generated from other plant species could activate these *Lt* MAPKs.

**Results:**

Our analysis found that just a 1-min exposure to GLV was enough to activate the *Lt* 46 kDa MAPK within 3 min and the 44 kDa MAPK within 15 min. This activation pattern showed similar kinetics to those observed after wounding, and the GLV and wound activated bands associated with these MAPKs displayed identical migration on sodium dodecyl sulfate polyacrylamide gels. Thirteen different commercially available plant volatiles (alcohols, aldehydes and ketones) were tested and all thirteen volatile compounds were able to activate these same *Lt* MAPKs. Furthermore, GLV derived from three other grass species as well as tomato, a dicot, were also shown to activate these MAPKs in *Lt*.

**Electronic supplementary material:**

The online version of this article (10.1186/s13104-017-3076-9) contains supplementary material, which is available to authorized users.

## Background

Plants in the field are faced with a variety of stresses on any given day. They must sense their environment, allocate and prioritize available resources to mobilize a coordinated response to alter their physiological and metabolic state in order to survive. Plants have evolved a variety of interconnected signaling networks and mechanisms to respond to these various types of stresses [1–3]. One of the major stresses forage and turf related grasses are subjected to is wounding. Unlike cereal grasses and many other crop species, forage and turf grasses are repeatedly cut or grazed upon throughout their lifecycle. Currently there is very little known concerning the perception, signalling or molecular responses associated with wound stress in grasses. When grasses are cut or damaged, they rapidly release a volatile chemical cocktail called green leaf volatiles (GLV) [4]. GLV and other plant volatiles have been shown to play a role in plant defense against insects and pathogens [5–9] and have been implicated to have a role in abiotic stress responses [9–11]. Furthermore GLV may prime or enhance the plant’s response to particular stresses and this priming effect may occur via inter- and/or intra-plant signalling [8, 9, 12–17].

Previously we found that mechanical wounding in six different grass species activated a 46 and 44 kDa mitogen-activated protein kinase (MAPK). The 46 kDa MAPK was found to be rapidly activated both locally and systemically in an adjacent unwounded tiller within 5 min of wounding in the model grass species *Lolium temulentum* (*Lt*) [18]. Similarly when leaf tissue was damaged by nonanoic acid, a nine-carbon-chain carboxylic acid used in grass herbicides, the 46 kDa MAPK was activated in the treated leaves within 5 min of exposure and in undamaged systemic tissues within 15 min [19]. Furthermore damaged caused by fire to *Lt* tiller leaf tips, activated both the 46 and 44 kDa MAPKs within 5 and 20 min respectively, and within 15 min in the systemic undamaged tiller [19].

MAPK signaling cascades have been shown to play a significant role in the regulation of defense, stress and developmental related pathways [20–22]. In response to a specific stress or environmental stimuli, MAPK signaling cascades, acting as an integral component of a coordinated signaling network, lead to the activation of specific downstream targets to elicit a stress specific response [22, 23]. The rapid activation of these MAPKs to multiple types of stresses in forage and turf grasses suggest that they play a significant role in the perception and response to these stresses [18, 19, 24]. Surprisingly in our investigation of the stress activation of MAPKs in *Lt*, we found that GLV released from grass clippings activated both the 46 and 44 kDa MAPKs [19]. The similar kinetics of activation of these MAPKs by GLV and by direct mechanical wounding indicate a potential role for these volatile compounds in wound signaling in forage and turf grasses.

In this manuscript we extend these studies to determine minimal exposure time necessary for GLV activation of the MAPKs. Additionally we tested 13 commercially available compounds associated with GLV to determine if a specific compound or class of compounds could activate the MAPKs. Lastly, we investigated if GLV from different plant species could activate the *Lt* MAPKs.

## Main text

### Methods

#### Plant materials

*Lolium temulentum* L. (*Lt*, Darnel ryegrass) cv. Ceres seeds were planted in 36 well flats, 2 × 1.5 × 2 in. well (1 plant/well) containing either Professional Growing Mix MM840 PC RSi or LA4 PC RSi (Sungro Horticulture, Canada). Plants were grown under 12 h photo-periods at 23 °C day and 18 °C night in Conviron E15, PGR15, PGV36 or PDW40 (Conviron, Winnipeg, Canada) growth chambers. Tissues used to generate GLVs were taken from *Lt*, *Brachypodium distachyon*, tall fescue, orchardgrass and tomato (cv Castlemart) plants grown from seed in 1 L pots in a greenhouse (24–28 °C day and 18 °C night). Plants were fertilized weekly using Jack’s Professional General Purpose 20-20-20 fertilizer (JR Peters Inc, USA).

#### Stress treatments

*Lt* receiver plants were grown in individual wells in flats in a growth chamber for 3–4 weeks. When the plants reached the two/three-tiller stage, the individual wells/pots were separated and the plants allowed to rest overnight prior to treatment.

Wounding of *Lt* plant was performed as described [18].

*Green leaf volatiles: Lt*, *Brachypodium*, tall fescue, orchardgrass or tomato leaf cuttings were placed on the bottom (approximately 0.5–1 in. deep) of three 8.3 L Nalgene multipurpose polycarbonate jars with covers (non air tight). The 1–2 in. long cuttings were crushed by hand and placed into the cylinders (with lids). Plants were exposed to vapors of 13 different volatile compounds (see Fig. 2a) by pipetting 10 or 25 µL of the compound onto a Q-tip in the cylinder. The cylinders containing the plant leaf clippings or volatile compounds were placed into a growth chamber under light for 5 min before adding plants. Into each cylinder three *Lt* plants were placed. The plants were treated and removed from the cylinders as previously described [19] at the time points indicated: Cylinder #1 time points 3, 15 and 30 min; Cylinder #2 time points 5, 20 and 45 min; Cylinder #3 time points 10, 25 and 60 min. The time course for the control plants was performed as described above except there were no grass clippings in the cylinders. Note for the 1 min exposure experiment, *Lt* plants were placed into the 3 cylinders as described above for only 1 min and then immediately removed from the cylinders. The cylinders containing the clippings were removed from growth chamber, while the plants remained in the chamber for the remainder of the time course. All plant tissues were collected, quick frozen with liquid nitrogen and stored at − 80 °C until processed. Sample preparation and immunoblot analysis was performed as described previously [18, 19, 24] and loading controls (Additional file 1: Figure S1).

### Results

**Fig. 1:**
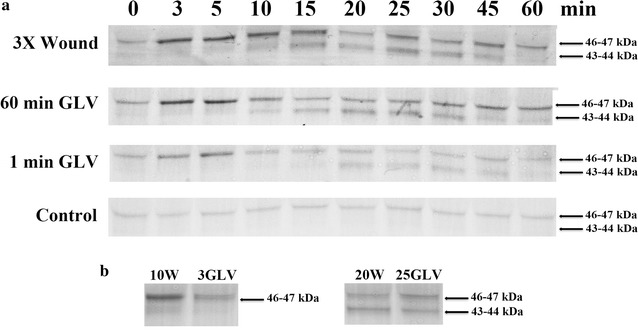
Exposure to green leaf volatiles activated the 46 and 44 kDa MAPKs in *Lt.* **a** Immunoblots showing the activation of the *Lt* MAPKs. 3X wound—*Lt* plants were wounded 3 times perpendicular across all tillers 1–2 in. apart, spaced vertically from the base of the plants, using a hemostat. Three-four week-old *Lt* plants at the two tiller stage were exposed GLV by placing them in a closed clear (non-air tight) cylinder with freshly cut and damaged *Lt* leaf tissue for the entire 60 min of the time course or for only 1 min. Control treatment plants were placed into the cylinders without any grass clippings present. **b** Immunoblots of *LT* plant extracts of time points taken from: 10W, 10 min after wounding; 3GLV, 3 min after GLV exposure; 20W, 20 min after wounding; 25GLV, 25 min after GLV exposure; were separated on adjacent lanes on a SDS-PAGE gel. One plant per time point was collected at the times indicated. To determine MAPK activity, immunoblots of protein extracts were performed using anti-phospho-MAPK (Erk1/2) antibody. The experiments were independently repeated three times. Representative blots are presented. Note while previously the MAPK bands were designated as 46 and 44 kDa MAPKs based on a comparison of their migration distance with protein molecular weight markers on SDS-PAGE gels, but their actual molecular weight range may be 46–47 and 43–44 kDa

This study expanded the analysis of GLV and their ability to activate MAPK signaling cascades in the model grass species *Lt*. Previously mechanical wounding was shown to rapidly activate a 46 and 44 kDa MAPK in *Lt* [18]. Similarly, exposure of *Lt* to freshly mowed grass clippings was also shown the activate MAPKs with similar kinetics [19]. The activation of the 46 and 44 kDa MAPKs in *Lt* by wounding and continuous exposure to GLV is shown in Fig. 1a. Interestingly, it takes only 1 min exposure to GLV from cut grass clippings to activate the MAPKs. The migration of the bands for the 46 and 44 kDa MAPK activated by wounding and GLV appear to be the same (Fig. 1b). Additionally, we investigated whether antibodies (Sigma, USA) to the *Arabidopsis* AtMPK3, AtMPK4 or AtMPK6, which have been shown to be involved in stress responses [25], would cross react with the *Lt* 46 or 44 kDa MAPK bands. While all 3 antibodies displayed cross-reactive bands in *Lt*, and the antibodies to AtMPK4 or AtMPK6 identified bands with very similar migration to the *Lt* anti-phospho-MAPK (Erk1/2) 46 kDa band, but they did not appear to be the same (data not shown).

**Fig. 2:**
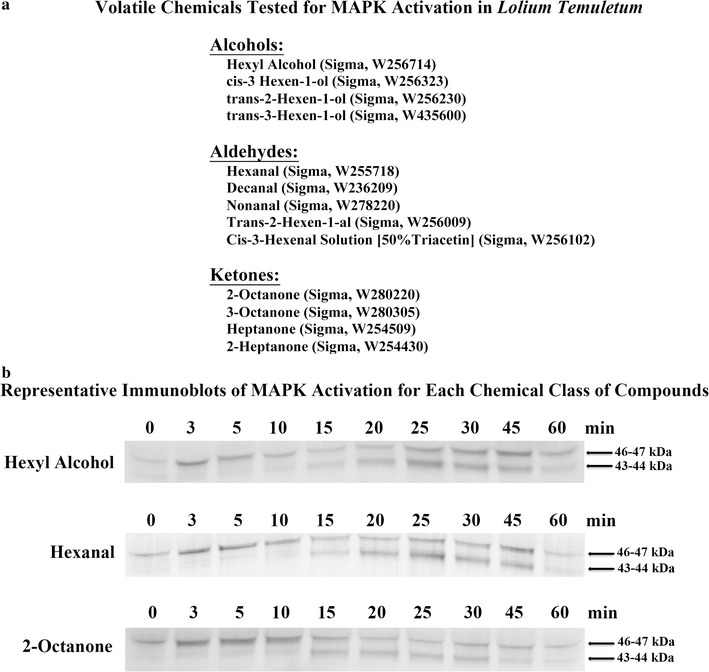
Plant volatile compounds activated the *Lt* 46 and 44 kDa MAPKs. **a** A list of 13 plant volatile compounds separated into different chemical groups (alcohols, aldehydes and ketones) that were tested for their ability to activate the *Lt* 46 and 44 kDa MAPKs. Chemical name (company, catalog number). **b** Representative immunoblots of MAPK activation for one member of each chemical class of compounds are presented. Plants were exposed to vapors of each individual volatile compound by pipetting 10 µL of the compound onto a Q-tip in the cylinder, and then 3–4 week-old *Lt* plants at the two tiller stage were placed into a closed clear (non-air tight) cylinder. One plant per time point was removed from the cylinder and collected at the times indicated. Note control treatment plants were placed into the cylinders without addition of a volatile compound—see Fig. 1. To determine MAPK activity, immunoblots of protein extracts were performed using anti-phospho-MAPK (Erk1/2) antibody. The experiments were independently repeated three times per compound

**Fig. 3:**
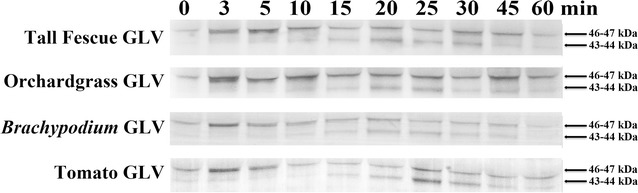
GLV generated from other plant species activated the *Lt* 46 and 44 kDa MAPKs. Three-four week-old *Lt* plants at the two tiller stage were placed into a closed clear (non-air tight) cylinder with freshly cut and damaged leaf tissue from plants indicated in the figure. One plant per time point was collected at the times indicated. Note control treatment plants were placed into the cylinders without any grass clippings present—see Fig. 1. To determine MAPK activity, immunoblots of protein extracts were performed using anti-phospho-MAPK (Erk1/2) antibody. The experiments were independently repeated three times. Representative blots are presented

GLV contain a wide range of volatile compounds [4, 26, 27], therefore we wanted to determine if a specific compound or class of compounds activated the *Lt* MAPKs. Thirteen commercially available compounds, many found in GLV [4], from 3 different chemical classes, aldehydes, alcohols and ketones were tested. Surprisingly all of the compounds tested (Fig. 2a) activated both the *Lt* 46 and 44 kDa MAPKs with some variation in kinetics. It should be noted that no significant differences for MAPKs activation were observed whether 10 or 25 µL of the compound was used. Since all the compounds activated both the 46 and 44 kDa MAPKs, and many of these compounds are present in other GLV released from other plant species, we wanted to see if other plant GLV could activate the MAPK in *Lt*. As shown in Fig. 3, when *Lt* was exposed to GLV from the orchardgrass and tall fescue forage grasses, the model grass *Brachypodium* or from the dicot tomato, all were found to have activated both the 46 and 44 kDa MAPK. For all specific GLV compounds and different plant species GLV tested, the *Lt* 46 kDa MAPK was activated within 3–5 min of exposure, and the *Lt* 44 KDa MAPK was activated after 15–25 min of exposure.

### Discussion

Plants utilize a variety of compounds to respond to their environment. In recent years volatile compounds have been implicated to have a role in a wide range of stresses. In this report we investigated further the activation of stress related MAPKs [18, 19, 24] in the model grass *Lt* by plant volatiles. For a signal to be effective it must be perceived in a short time and initiate a rapid response. Our analysis found that just a 1-min exposure to GLV was enough to activate the *Lt* 46 kDa MAPK within 3 min and the 44 kDa MAPK within 15 min. This activation shows similar kinetics to those observed after wounding, and the GLV and wound activated bands associated with these MAPKs had nearly identical migration on sodium dodecyl sulfate polyacrylamide gel electrophoresis (SDS-PAGE). Our analysis also found that 13 different commercially available plant volatiles, many of which were found to be released by tufted hairgrass after mechanical wounding [4], were also able to activate these same MAPKs. Furthermore, GLV derived from three other grass species as well as tomato, a dicot, were also able to activate these MAPKs. These results indicate that this MAPK activation is a generalized response to the release of volatiles associated with plant damage. However, not all plant derived volatiles associated with the plant’s wound response activated these MAPKs. Previously, we showed that the direct exposure of *Lt* plants to the volatile signaling molecule methyl jasmonate (MJ) did not activate the 46 and 44 kDa MAPKs, however when plants were exposed to MJ prior to wounding, it did enhance the wound activation of the 44 kDa MAPK [18]. Synergistic effects between GLV and jasmonic acid have been observed in other plant species [28]. Furthermore *Lt* plants treated with linolenic acid, the precursor to JA were unable to activate either MAPK [19]. The GLV and volatile compounds released by plants after damage may be involved in both inter- as well as intra-plant signaling. In the field plants are often being exposed to below threshold levels of volatile compounds for activation of a stress response. However this intermittent low level exposure may prime or sensitize the plant, providing a more rapid and robust response towards potential future stresses [8, 9, 12–17]. The ability of grasses to perceive these compounds in their surrounding environment and to prime the plant’s response for the potential oncoming wound stress could be advantageous for grasses, which are routinely cut and grazed upon throughout their lifecycle. While other types of signals, such as reactive oxygen species [29, 30], are used for intra-plant signaling to undamaged systemic tissue, volatile compounds have been implicated to play a more significant role in plant species with less developed vascular systems [31]. The data presented in this report suggests links between GLV and wound signaling. Future research will be directed at determining what downstream elements are being activated by exposure to GLV and wounding, how they are connected, and how the plant responds at the molecular or physiological level to these different stimuli.

## Limitations

Concentration curves for the various commercially available compounds for MAPK activation were not done. Since so many compounds were tested, it made it extremely difficult to directly compare intensity of MAPK activation between samples, however kinetics (timing) of the activation the 46 and 44 kDa MAPKs was possible. The actual concentrations of GLV in the field that forage or turf grasses are exposed to when they are cut, as in a lawn or for collection of hay, are unknown. However cut grass will release high levels of GLV that can be detected at great distances suggesting that in localized areas GLV concentrations could approach those used during our experiments.

## Additional file

**Additional file 1: Figure S1.** Protein loading controls and positive–negative control strip for antibody. A) In order to confirm the equal loading of protein per lane (50 µg/lane) on the 10% SDS-PAGE gels and to verify the accuracy of the BCA protein assay, duplicate gels were run for randomly selected sample preps and were stained with coomassie blue. As shown stained gels for samples, *Lt*- 60 min GLV treatment and *Lt*- exposed to 2-octanone. Furthermore, these two same sample preps were then separated on SDS-PAGE gels and the separated proteins transferred to Immobilon P and the blots stained with coomassie blue to confirm efficiency of transfer. It should be noted that periodically during our analyses, the consistency of protein transfers to Immobilon P membranes were checked and confirmed by staining randomly selected membranes with ponceau S prior to the immunoblot analysis. B) Example of Immobilon P control strip to assess antibody activity; C: negative control—untreated *Lt* tissue 0 min; W: positive control—*Lt* 10 min post wounded tissue. This control strip was added to the immunoblot analyses as an internal quality control to assess antibody sensitivity and activity.

## Abbreviations

ERK: extracellular signal regulated kinase
GLV: green leaf volatiles
JA: jasmonic acid
*Lt*: *Lolium temulentum*
MAPK: mitogen-activated protein kinase
MJ: methyl jasmonate
PAGE: polyacrylamide gel electrophoresis
SDS: sodium dodecyl sulfate
