# Supplementary figures and images for: Activation of MAP kinases by green leaf volatiles in grasses
Source: BMC Res Notes. 2018 Jan 29;11:79. doi: 10.1186/s13104-017-3076-9 (PMC5789745; doi:10.1186/s13104-017-3076-9)

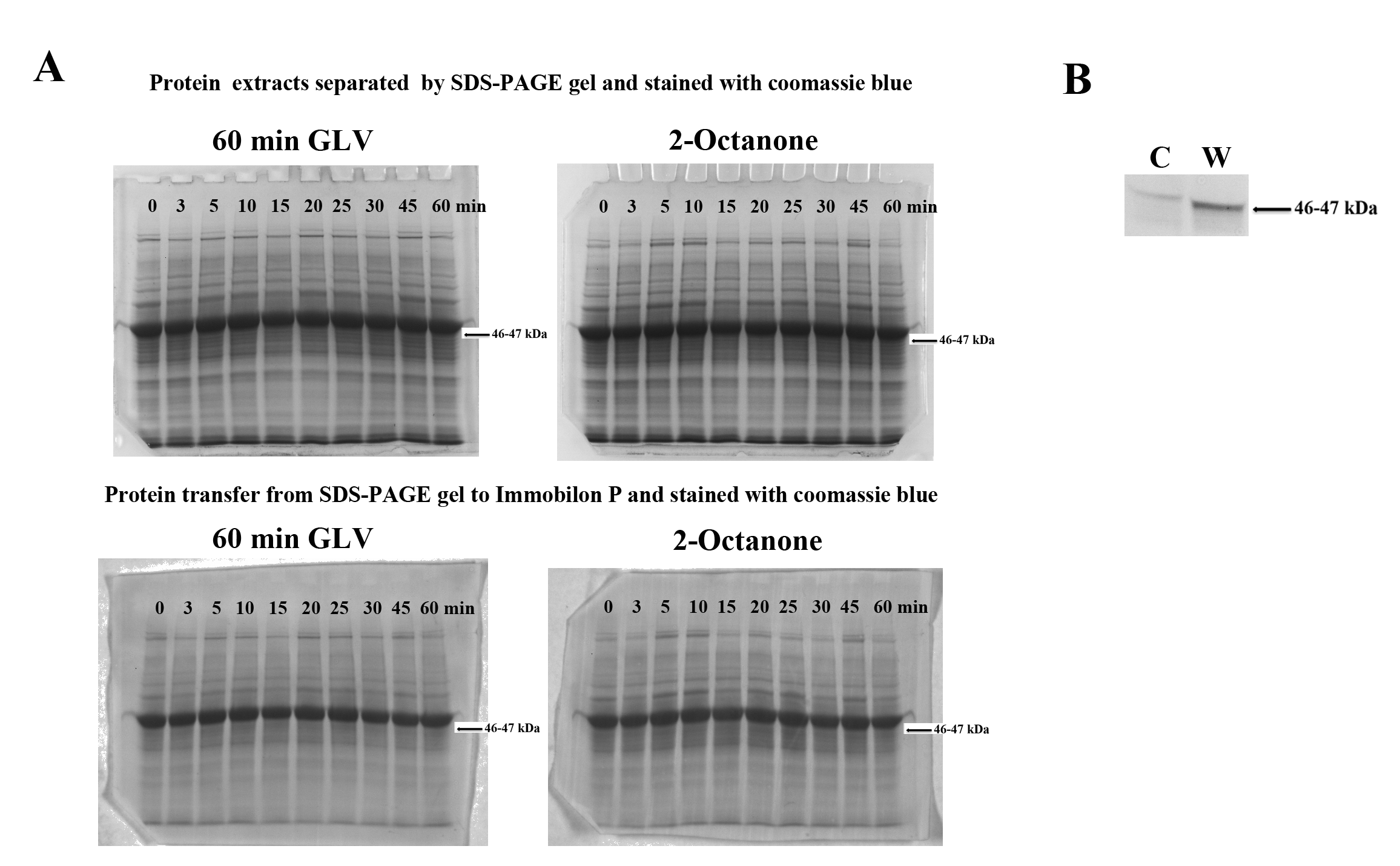

Supplement: Supplementary file 1 — Additional file 1: Figure S1. Protein loading controls and positive–negative control strip for antibody. A) In order to confirm the equal loading of protein per lane (50 µg/lane) on the 10% SDS-PAGE gels and to verify the accuracy of the BCA protein assay, duplicate gels were run for randomly selected sample preps and were stained with coomassie blue. As shown stained gels for samples, Lt- 60 min GLV treatment and Lt- exposed to 2-octanone. Furthermore, these two same sample preps were then separated on SDS-PAGE gels and the separated proteins transferred to Immobilon P and the blots stained with coomassie blue to confirm efficiency of transfer. It should be noted that periodically during our analyses, the consistency of protein transfers to Immobilon P membranes were checked and confirmed by staining randomly selected membranes with ponceau S prior to the immunoblot analysis. B) Example of Immobilon P control strip to assess antibody activity; C: negative control—untreated Lt tissue 0 min; W: positive control—Lt 10 min post wounded tissue. This control strip was added to the immunoblot analyses as an internal quality control to assess antibody sensitivity and activity. [file 13104_2017_3076_MOESM1_ESM.tif]
